# ONE QUALITY concept: a narrative perspective to unravel nutritional challenges, controversies, and the imperative need of transforming our food systems

**DOI:** 10.3389/fnut.2024.1379159

**Published:** 2024-04-15

**Authors:** Roberto Menta, Ginevra Rosso, Federico Canzoneri

**Affiliations:** Soremartec Italia Srl, Ferrero Group, Alba, Italy

**Keywords:** ONE QUALITY, database, Med Diet, SFA, plant-based, food system, NCDS

## Abstract

Ensuring a healthy and sustainable diet for all should be a global priority, and to achieve this goal the food system requires substantial changes. Adopting a one-size-fits-all approach is not feasible, and we need to consider the cultural particularities of each geography and not try to export models that work in one place but may be unsustainable in others. Our discussion will center on two key aspects within this overarching process: (a) the combination of a rigorous evidence-based approach with existing or proposed Nutritional Guidelines and policies required to realize the “ONE HEALTH” and “ONE QUALITY” concepts. Examining the Mediterranean diet and the latest findings on saturated fats will aid us in comprehending the necessary paradigm shift required to formulate new guidelines with substantial impact in preventing the rising prevalence of Non-Communicable Diseases worldwide; (b) the adequacy and scope of the data bank necessary to develop a global, science-based approach.

## 1 Introduction

A perfect storm is currently brewing within the global food system. Not only diet has an effect on health; factors such as population growth, climate change, inequalities among different regions and individuals, urbanization, and evolving cultural values regarding food understanding and use have created a complex and multifaceted reality that affects us all (1–3). Sustainable and healthy diets have become the guiding principles of prominent institutions like the Food and Agriculture Organization (FAO) and the World Health Organization (WHO) (4). The role of food in determining health outcomes is gaining importance at the citizen level, leading to the emergence of new dietary habits. The intricacies and interrelationships among the various components of the food system do not permit definitive and conclusive solutions. The growing significance of exposome research (5) represents one of the few holistic approaches currently embraced. For instance, we consider whether urbanization plays a more or less critical role than physical activity levels and how these factors impact the ultimate outcomes. Furthermore, the role of gender has been significantly underestimated, and evidence of its influence is poised to unveil new perspectives in nutrition research and policy (6). However, in light of the growing awareness aimed at resolving these challenges, it is high time to initiate an inclusive and comprehensive debate. Assuming that a Healthy Diet is, and will remain, a fundamental prerequisite, we will begin by defining this essential aspect of human heritage and basic need.

## 2 Healthy diet: an expanding pillar inside food system

There are numerous definitions of a healthy diet. In this discussion, we will adopt and expound upon the definition recently provided by Neufeld et al. (7) that states, “*A healthy diet is health-promoting and disease-preventing. It provides adequacy, without excess, of nutrients and health-promoting substances from nutritious foods and avoids the consumption of health-harming substances…*”

Therefore, the concept of a “healthy diet” is inherently linked to food safety. Once food security and food safety are assured, along with food availability, we can work toward achieving the goal of consistent food quality for everyone. While this concept may appear self-evident, there remains a considerable journey ahead to achieve it on a global scale. Despite acknowledging that food safety is a collective responsibility involving various stakeholders, the persistence of unsafe foods and the inadequate handling of food safety incidents hinders its global assurance (8). The ONE QUALITY concept is prevalent in several ongoing projects at the level of major UN agencies such as WHO and FAO. It is increasingly recognized as a strategic approach to promoting a healthy diet for all.

With these principles in mind, let us explore a few examples of the uncertainties that arise in real-life situations, such as the Mediterranean Diet (Med Diet) and fat controversy and their ripple effects on diet, nutritional health, and the entire food system.

The Med Diet has evolved into a kind of “panacea” and a model for a healthy diet. Ancel Keys’ renowned “Seven Countries Study” (9) introduced a somewhat vague concept based on a self-approved synthesis of observational data. This was during a period when the economic situation in the Mediterranean region was quite poor, and the collected data were somewhat arbitrarily selected. The tremendous popularity that followed the publication of Keys’ work elevated the Med Diet to the status of a global paradigm.

The understanding and adoption of the Seven Countries Study concept regarding diet composition revolved around the idea that saturated fats promote an increase in blood cholesterol levels, which in turn leads to cardiovascular diseases (CVDs). However, this interpretation was not exactly what A. Keys himself proposed. He argued that despite the significant and fundamental role of diet, it was not the sole factor at play. Keys (9) emphasized the importance of factors such as physical activity, strong family ties, and a relaxed way of life as primary components and determinants of better health. In essence, A. Keys anticipated the concept of lifestyle and believed it was responsible for the lower incidence of CVDs.

Over time, the positive effects of the Med Diet have been attributed to various factors, including olive oil (OO - Extra virgin: EVOO), low meat protein consumption, and, more recently, the content of polyphenols (10).

Now, let us consider a hypothetical scenario where the Med Diet is adopted as a universal approach. Several critical and unrealistic points become evident:

OO, and even less so EVOO, cannot serve as the universal oil. The annual production of 2–3 million tons of olive oil represents approximately 1% or less of the total oils and fats needed globally (11). Simply put, there is insufficient olive oil and olives to meet the demands of more than a few countries. Therefore, alternative oils should be identified.

The preference for low meat and relatively high fish intake should align with the accepted data that sea-based protein sources constitute only about 6% of the world’s protein needs. While aquaculture may increase fish protein availability in the future, there are several sustainability concerns associated with this practice that require thorough evaluation.

More recently, “polyphenols” have been suggested to have protective effects and promote healthier dietary and metabolic habits. However, complete information on the metabolism, absorption, and long-term effects of polyphenols is lacking in the scientific literature, with only a few exceptions. The Phenol-Explorer, for example, cites data on a total of 458 foods and 501 different polyphenols (12). There are several million, if not billions, of food items, while polyphenols constitute a family of phytochemicals with more than twenty thousand identified molecules (13). Furthermore, data on polyphenol metabolism are sparse and not systematically explored (14). With such limited information available, it becomes challenging to make any general inferences about the effects of this important dietary component in a science-based approach.

While the Mediterranean Diet promotes low meat protein consumption, comparing it with the Indian diet—the largest vegetarian diet worldwide—yields compelling evidence for a more robust comparison. There are various models of vegetarian diets globally, and the Indian diet stands out as one of the most frequently cited and recognized. When we account for the impact of scarcity and poverty, specifically addressing inadequate nutrient and calorie intake, the positive health effects of the traditional Indian diet become evident (15). However, these statements require careful evaluation due to several confounding factors, including economic, geographic, and cultural influences.

- Vegetable oil: The Indian diet utilizes groundnut and mustard oil instead of olive oil (16).
- Fish is not a standard or significant component of the Indian diet, while legumes are the preferred and more relevant source of proteins.
- Omega-3 fatty acids (ω3) in the Med diet primarily come from fish, whereas in the Indian diet, they come from vegetable oils.

These differences raise the question: Can both diets be compared, given their different sources of macronutrients and completely different chemical compositions? Even if we accept this premise -which may not necessarily be the case- where does the scientific evidence stand when we compare the nutrients of these diets?

On top of that, it is well-known that there is significant variability in the fatty acid components of several vegetable oils (17). For instance, in OO the concentration of palmitic acid can range from 7.5 to 20%, and the oleic acid concentration can vary from 55 to 83% (18).

**Figure 1:**
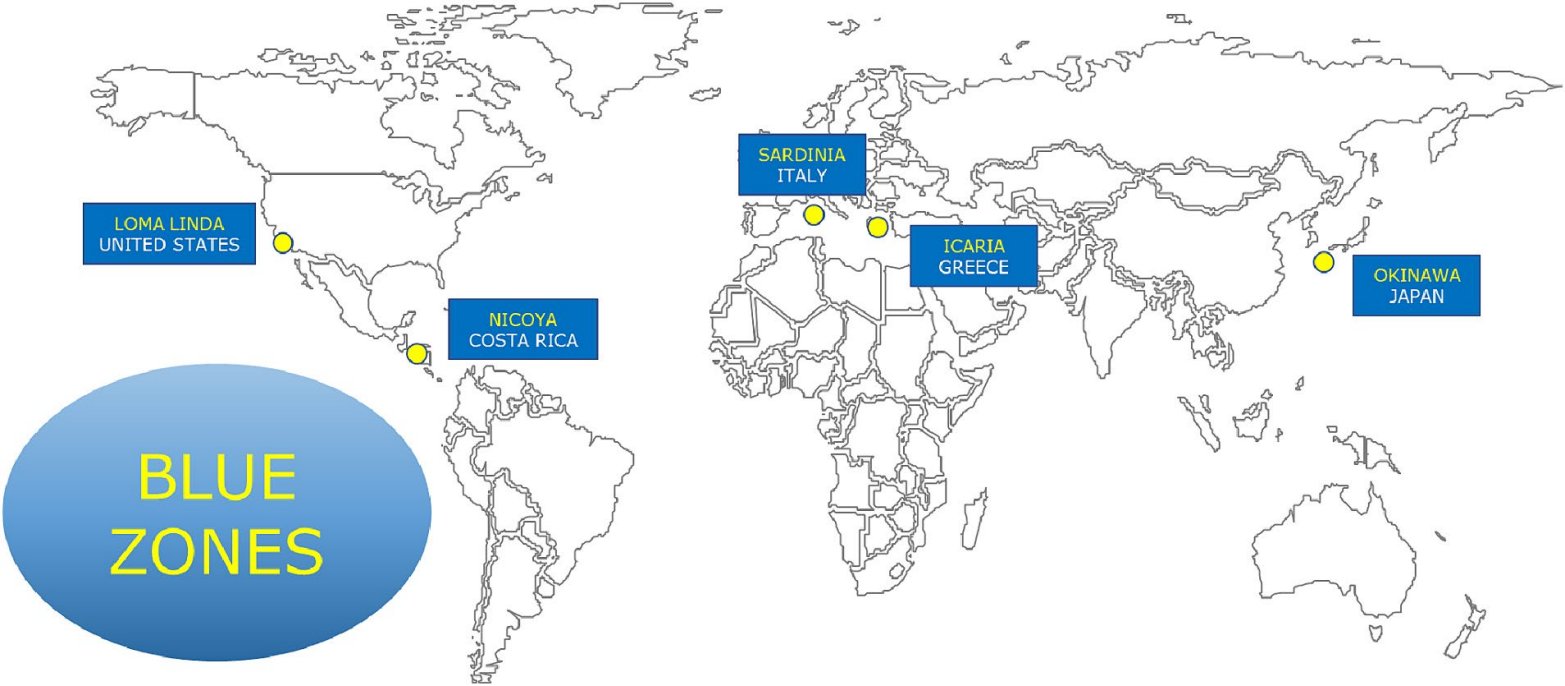
Blue zones are regions where a higher than usual number of people live much longer than average. There are five blue zones areas in the world.

Additional insights can be gleaned from the “Blue Zones” (Figure 1). There are several points of overlap between some Blue Zone diets and the Med Diet, but there are also significant differences. For example, in the Sardinia Blue Zone diet, the primary source of animal protein is sheep, while the Med Diet emphasizes fish protein. The Okinawa Blue Zone diet includes a substantial portion of pig components. Calorie restriction cannot be considered a universal factor among these Blue Zone diets, as the caloric intake varies significantly, ranging from 1,500 kcal/day in Ikaria, Greece, to 2,392 kcal/day in Nicoya, Costa Rica (19).

When we compare the Ikaria’s diet to the Western-style diet in the United States, it’s easy to appreciate the significant differences, particularly in the amount of fat as an energy source (see Table 1).

**Table 1:** It presents evidence highlighting three main differences between the average diet of Americans and that of Ikaria: daily calorie intake, the percentage of energy derived from carbohydrates, and the percentage of energy derived from fats (20, 21).

	Total Energy	Carbohydrate % of daily calories	Protein % of daily calories	Fat % of daily calories	Alcohol % of daily calories	Fiber grams/day
Average American adult	2,270	43%	16%	36.5%	4.5%	14 g
USDA ideal American adult	2,240	53%	16%	27%	4%	32 g
Ikaria, Greece	1800*	32%	13%	50.5%	4.5%	24 g

*Amount adjusted for age and sex of average population.

If we compare the amount of fat recommended by dietary guidelines, we can see that the Ikaria Blue Diet provides approximately 100 grams of fat per day, while the average intake in the United States is around 90 grams per day. Furthermore, when we analyze the Sardinia Blue Zone diet, we find that the predominant source of dietary fat is of animal origin, and a significant portion of it is saturated. This pattern holds true for other Blue Zones as well (22).

Available data suggest different and very plausible interpretations. The calorie amount might be part of the explanation for the positive effects of the Med Diet recorded in the Seven Countries Study. However, the primary role of saturated fatty acid (SFA) is being challenged by new epidemiological analyses and meta-analyses, including valuable contributions like the Cochrane reviews. Notably, the hypothesis of a positive role, without the biological identification of related mechanisms, remains lacking.

A fresh start in analyzing and interpreting the available data seems imperative, as well as the development of new dietary guidelines that should be rigorously evidence-based.

## 3 Fat controversy, the role of saturated fatty acids

The discussion surrounding the role of SFA in dietary guidelines has been ongoing. In 2010, a meta-analysis (23) challenged the link between SFA intake and NCDs. P.W. Parodi’s study (24), synthesizing data from major population-based studies, revealed a weak positive correlation, contradicting the notion of a negative relationship between SFA intake and daily energy intake.

Randomized controlled trials (RCTs), considered the gold standard in nutritional research, were pivotal in understanding SFA’s role. D. Mozaffarian’s seminal paper initiated the scientific discourse (25), but the Cochrane group critically evaluated it, identifying biases in data treatment, particularly regarding industrially trans-fatty acids (iTFA) and psychoactive drugs (26). Their findings questioned the efficacy of replacing SFA with polyunsaturated fatty acids (PUFA), highlighting confounding factors from iTFA (27).

The “fat controversy” today stems from varied interpretations of cholesterol, low-density lipoprotein (LDL), and high-density lipoprotein (HDL). The perspective presented emphasizes two fundamental principles: first, the consideration of evolution, and second, the recognition that human metabolism, rooted in biochemistry, offers more robust and evidence-based insights than mere epidemiological associations, keeping in mind that this kind of studies are the starting point to find causal links between diet and health.

Exist extensive discussions on the role and level of evidence from epidemiological studies, cohort studies, and RCTs in defining dietary guidelines, particularly regarding saturated fats (28, 29). The focus shifts to the biochemical evidence, guided by Bill Lands’ understanding of fat metabolism through the Lands’ cycle framework (30). Emphasizing the significance of plasma LDL, the paper suggests that the causal role of LDL in inflammation is debatable, with excess energy intake being the primary driver. Metabolism adaptations, assessed through β-hydroxybutyrate levels, are considered in the context of evidence.

The fatty acid composition of any tissue is highly variable, this highlights the role of Highly Unsaturated Fatty Acids (HUFA) and the causative role of oxidized fats in chronic low-grade inflammation, leading to cardiovascular diseases (CVDs) (31). Acknowledging contradictory data, we believe that a good starting point would be that unbalanced PUFA intake may trigger inflammation due to competition in metabolic cascades between Omega-3 (Ω3) and Omega-6 (Ω6), favoring an excess of Ω6, causing metabolic imbalance.

A key convergence point is that SFA intake is not the direct cause of atherosclerosis and related NCDs. Longevity, as demonstrated by Blue Zones, suggests diverse approaches. We advocate a shift from a nutrient-focused to a holistic dietary concept, considering the entire diet’s effects.

In the context of ongoing climate change affecting food composition, there’s a call for adaptable food systems. Regulatory bodies are urged to adopt a flexible approach, acknowledging data limitations and being open to updating dietary guidelines. Saturated fats, in particular, are highlighted as an example, with prominent scientists advocating for changes in dietary guidelines, especially regarding the role of saturated fats (32).

## 4 The database used for food composition by policymakers and operators is far from being readily available

The role of the scientific database is crucial for defining the best possible Dietary Guidelines. Currently, they are weak and inconsistent. We align with those who support the idea that “something – limited evidence: authors’ comment – is not better than nothing” (33). While this statement pertains to energy intake, it can be extended to other biological entities.

Considering the complexity of the evolving Food System and the crucial role of Dietary Guidelines, there is unanimous agreement that the starting point, i.e., the food composition database, should be complete and up-to-date. Are the current food databases of the required and expected quality? Furthermore, do the levels of detail provided in these databases align with the latest knowledge in the field of nutritional research and policies? In a perspective of Aleta et al. (34) emphasize the critical step of characterizing exposure to the food system in the context of sustainable and healthy nutrition research. The primary challenge identified is obtaining comprehensive compositional data that encompass various aspects such as nutrient content, bioactive compounds, energy density, bio-accessibility and bioavailability of nutrients, together with sensory attributes, processing methods, socio-economic and environmental impacts. Despite efforts by initiatives like EuroFIR, USDA, and INFOODS, existing data are often heterogeneous, noisy, and incomplete.

Using olive oil as an example, the paper (34) illustrates the challenges related to data quantity and quality in nutritional studies. Olive oil composition, impacted by genetic, environmental, and technological factors, varies widely. Data incompleteness, leading to misleading results or unsubstantiated correlations, hampers progress. Traditional nutritional approaches focusing on main components neglect non-trivial interactions and chemical components not in the database, influencing results. It is highlighted the need for increased quality and availability of food chemical composition data, emphasizing the difficulty in establishing meaningful causal associations without comprehensive information. Variability associated with intake estimations is considered multidimensional, involving economic capacity, cultural factors, and social influences. Defining relations between nutrition, health status, and the production system requires a system-wide analysis, posing a pressing challenge for providing global dietary recommendations that consider cultural and behavioral covariates.

Scientifically speaking, derivatives that inform nutritional guidelines cannot be supported if they focus only on macronutrients. Despite our conviction of their absolute necessity for dietary guidelines, we can collectively agree that the available data are at least very unsatisfactory. In other words, while we all agree on the concept of a “Healthy Diet,” the definition of quality requires the inclusion of new available biomarkers and, even more, new hypotheses and theories.

Today, a strong trend supports a shift from our westernized diet to a more, or substantially more, plant-based diet (35). Increasing the amount of vegetable protein at the expense of fats and carbohydrates, mainly reducing simple carbohydrates, is universally accepted for better health and a less or not relevant impact on the environment. If we follow this trend, it is time to search for the best possible treatment of plant-based food to achieve both goals of better health and a safer planet.

## 5 Discussion

Globally, the primary determinants of food choice are taste and price. While the food industry can now offer products that closely align with dietary recommendations, their success varies significantly. Taste, however, remains a critical factor. Interestingly, taste preferences can be modulated or evolve with age.

The challenge for our food system is to create products that are in line with current scientific knowledge, while also considering the effort required to achieve the same sensory excellence using alternative and environmentally friendly ingredients. Balancing risk and communication to consumers is crucial relying on low or very low levels of evidence may breed skepticism, while demanding large investments with high risks may not yield the desired results.

We all need to join efforts, ideas, investments, and thoughts to change the food system. Our proposal is to (re)start from a common shared level: evidence-based practices. Governments must show leadership in adopting and implementing food safety policies that ensures that every stakeholder knows and properly plays its role, from prevention to response otherwise, access to safe food for all will remain an elusive goal. Plant-based foods are and will be a major component of the human diet. We should learn from the unsuccessful approaches adopted in the past, avoiding dogmatic positions such as the one related to SFAs, which are no longer sustainable as the major responsible for CVDs. As the most authoritative scientists have claimed for many years, the drivers are and will be international agencies such as WHO, FAO, and regulatory bodies. They should define the need to demonstrate and promote the advantages of shifting from today’s “normality” to the future constraints that environmental degradation imposes on all of us.

We have the concrete chance to write a new chapter in food development, the food market, but the only possible way is to define a shared concept of ONE QUALITY for all, everywhere, as the basis for any further discussion.

## Data availability statement

The original contributions presented in the study are included in the article/supplementary material, further inquiries can be directed to the corresponding author/s.

## Author contributions

RM: Conceptualization, Writing – original draft. GR: Writing – review & editing. FC: Writing – review & editing, Visualization.
